# Limited impact of the COVID-19 pandemic on colorectal cancer care in the Netherlands in 2020

**DOI:** 10.1007/s00384-022-04209-4

**Published:** 2022-08-19

**Authors:** Joyce Meijer, Marloes A. G. Elferink, Geraldine R. Vink, Femke P. C. Sijtsma, Jeroen Buijsen, Iris D. Nagtegaal, Pieter J. Tanis, Miriam L. Wumkes, Ignace H. J. T. de Hingh, Sabine Siesling, On behalf of the COVID and Cancer-NL Consortium

**Affiliations:** 1 Department of Research and Development, Netherlands Comprehensive Cancer Organisation (IKNL), Godebaldkwartier 419, 3511 DT Utrecht, the Netherlands; 2 Department of Medical Oncology, University Medical Center Utrecht, Utrecht University, Heidelberglaan 100, 3584 CX Utrecht, the Netherlands; 3 Department of Radiation Oncology (Maastro), GROW School for Oncology, Maastricht University Medical Center, 6202 AZ Maastricht, the Netherlands; 4 Department of Pathology, Radboud University Medical Center, Geert Grooteplein Zuid 10, 6525 GA Nijmegen, the Netherlands; 5 Department of Oncological and Gastrointestinal Surgery, Erasmus Medical Centre, Dr. Molewaterplein 40, 3015 GD Rotterdam, the Netherlands; 6 Department of Medical Oncology, Jeroen Bosch Hospital, Henri Dunantstraat 1, 5223 GZ ’s-Hertogenbosch, the Netherlands; 7 Department of Surgery, Catharina Cancer Institute-Catharina Hospital, Michelangelolaan 2, 5623 EJ Eindhoven, the Netherlands; 8 Department of Health Technology and Services Research, Technical Medical Centre, University of Twente, Drienerlolaan 5, 7522 NB Enschede, the Netherlands

**Keywords:** Colorectal cancer, COVID-19, Nationwide, Pandemic, Treatment

## Abstract

**Purpose:**

The COVID-19 pandemic had a major impact on the health services worldwide. We aimed to investigate the impact of the pandemic on colorectal cancer (CRC) care in the Netherlands in 2020.

**Methods:**

CRC patients, diagnosed in 2018–2020 in the Netherlands, were selected from the Netherlands Cancer Registry (NCR). The year 2020 was divided in four periods reflecting COVID-19 developments in the Netherlands (pre-COVID, 1st peak, recovery period, 2nd peak) and compared with the same periods in 2018/2019. Patient characteristics and treatment were compared using the Chi-squared test. Median time between diagnosis and treatment, and between (neo)adjuvant therapy and surgery were analyzed by the Mann–Whitney *U* test.

**Results:**

In total, 38,021 CRC patients were diagnosed in 2018/2019 (*n* = 26,816) and 2020 (*n* = 11,205). Median time between diagnosis and initial treatment decreased on average 4 days and median time between neoadjuvant radiotherapy and surgery in clinical stage II or III rectal cancer patients increased on average 34 days during the three COVID-19 periods compared to the same periods of 2018/2019. The proportion of colon cancer patients that underwent elective surgery significantly decreased with 3.0% during the 1st peak. No differences were found in the proportion of patients who received (neo)adjuvant therapy, systemic therapy, or no anti-cancer treatment.

**Conclusion:**

Only minor changes in the care for CRC patients occurred during the COVID-19 pandemic, mostly during the 1^st^ peak. In conclusion, the impact on CRC care in the Netherlands was found to be limited. However, long-term effects cannot be precluded.

## Introduction

The coronavirus disease 2019 (COVID-19) pandemic, known as severe acute respiratory syndrome coronavirus 2 (SARS-CoV-2), had a major impact on healthcare services worldwide [1]. After the first confirmed COVID-19 infection in the Netherlands on February 27, 2020, the coronavirus spread rapidly through the country [2]. The Dutch official authorities have subsequently taken various societal measures and healthcare institutions took precautionary measures to reduce the further spread of COVID-19 [3]. 

The Dutch population was advised to only visit a general practitioner (GP) with acute complaints. Moreover, national screening programs were suspended, including the screening program for colorectal cancer (CRC), which was halted from mid-March 2020 to mid-May 2020 [4, 5]. Subsequently, a decrease in CRC diagnoses and CRC surgical procedures was seen due to a lower number of primary care referrals and fewer individuals invited for Faecal Immunochemical Testing (FIT) screening than usual [6–8]. In the second part of 2020, a catch-up in CRC diagnoses was observed since the Dutch government decided to expand the screening colonoscopy capacity up to 120% [9]. Previous analyses, with data available from 25 hospitals until June 2020 (week 26), found a limited impact of the COVID-19 pandemic on CRC care in the Netherlands for the first half of 2020 [10].

The impact of the COVID-19 pandemic on national CRC care in the Netherlands for the whole year of 2020 is unknown. Therefore, the aim of this study was to investigate the impact of the COVID-19 pandemic on CRC care in the Netherlands during 2020 with national data.

## Methods

### Data collection

Data were selected from the Netherlands Cancer Registry (NCR), which registers all newly diagnosed malignancies based on notification by the Nationwide Pathology Databank (PALGA) since 1989 and is hosted by the Netherlands Comprehensive Cancer Organisation (IKNL). Information on patient and tumor characteristics, treatment, and diagnosis is registered from medical records by data managers of the NCR. Topography and morphology are coded using the International Classification of Diseases for Oncology [11]. TNM classification (8th edition) was used for staging the tumor [12]. The Eastern Cooperative Oncology Group (ECOG) Scale of Performance Status (PS) was used to classify performance status.

### Patients and definitions

Patients ≥ 18 years diagnosed with CRC in the period 2018–2020 were included in this study.

Age at diagnosis was grouped into ages < 55, 55–75, and > 75 years.

The year 2020 was split into four periods reflecting COVID-19 developments in the Netherlands [3]: pre-COVID period (January–February), 1st peak (March–May), recovery period (June–September), and 2nd peak (October–December). Patients were stratified according to these four periods based on date of diagnosis. To analyze the proportion of patients receiving adjuvant therapy and time between surgery and start of adjuvant therapy, patients were divided in these four periods based on date of surgery.

### Statistical analysis

Analyses were done separately for colon cancer and rectal cancer, except for analyses regarding stage IV patients. All analyses compared the four COVID-19-related periods of 2020 to the same periods of 2018 and 2019 combined.

Analyses regarding neoadjuvant therapy were done for patients with clinical stage II or III rectal cancer, and regarding adjuvant therapy for patients with stage III colon cancer. Proportions of patients who underwent a resection or who received a stoma were determined for stage I–III patients: local surgery consists of endoscopic resection, transanal endoscopic microsurgery, or transanal minimally invasive surgery. Finally, proportions of patients who were treated with systemic therapy (with or without another treatment) or who did not receive anti-cancer therapy were calculated for stage IV patients. Differences in baseline characteristics and treatment were tested using the Chi-squared test.

Median time intervals (in days) were calculated between diagnosis and initial treatment, between start of neoadjuvant therapy and surgery, and between surgery and start of adjuvant therapy. Differences in median time intervals were tested using the Mann–Whitney U test.

StataSE version 17 software was used to analyze all data and for all tests performed, a two-sided p-value of < 0.05 was considered statistically significant. The p-values were calculated excluding missing values.

## Results

### Population

In total, 38,021 patients were included. A total of 19,948 colon cancer patients were diagnosed in 2018/2019 and 8216 in 2020, and the number of rectal cancer patients in these years were 6868 and 2989, respectively. The baseline characteristics are presented in Table 1 (colon cancer) and Table 2 (rectal cancer).

**Table 1:** Characteristics of patients diagnosed with colon cancer by periods of 2020 versus 2018/2019

		January–February		March–May		June–September		October–December	
			Pre-COVID		1st peak		Recovery		2nd peak
		2018/2019	2020	2018/2019	2020	2018/2019	2020	2018/2019	2020
		*N* (%)	*N* (%)	*N* (%)	*N* (%)	*N* (%)	*N* (%)	*N* (%)	*N* (%)
Total		3476	1472	5115	1756	6534	2783	4823	2205
Age	< 55 years	269 (8)	130 (9)	463 (9)	189 (11) *	591 (9)	270 (10) *	437 (9)	201 (9)
	55–75 years	1809 (52)	723 (49)	2711 (53)	850 (48)	3270 (50)	1240 (45)	2410 (50)	1108 (50)
	> 75 years	1398 (40)	619 (42)	1941 (38)	717 (41)	2673 (41)	1273 (46)	1976 (41)	896 (41)
Gender	Male	1838 (53)	772 (52)	2688 (53)	915 (52)	3331 (51)	1419 (51)	2480 (51)	1061 (48) *
	Female	1638 (47)	700 (48)	2427 (47)	841 (48)	3203 (49)	1364 (49)	2343 (49)	1144 (52)
PS	0	1053 (59)	526 (60)	1600 (60)	578 (57) *	2074 (59)	874 (53) *	1584 (59)	803 (61)
	1	527 (30)	236 (27)	794 (30)	277 (27)	1011 (29)	519 (31)	749 (28)	366 (28)
	2–4	197 (11)	112 (13)	294 (11)	160 (16)	437 (12)	266 (16)	340 (13)	158 (12)
	Unknown	1699	598	2427	741	3012	1124	2150	878
Stage	I	939 (28)	386 (27)	1338 (27)	367 (21) *	1696 (27)	620 (23) *	1257 (27)	541 (25)
	II	839 (25)	372 (26)	1308 (26)	429 (25)	1688 (26)	747 (27)	1189 (25)	600 (28)
	III	903 (27)	346 (24)	1294 (26)	416 (24)	1652 (26)	708 (26)	1246 (26)	540 (25)
	IV	703 (21)	333 (23)	1048 (21)	507 (29)	1343 (21)	647 (24)	1029 (22)	479 (22)
	Unknown	92	35	127	37	155	61	102	45

*P*-values were calculated using Chi-squared tests, asterisks indicate statistically significant differences (*p* < 0.05)

*PS* performance status

**Table 2:** Characteristics of patients diagnosed with rectal cancer by periods of 2020 versus 2018/2019

		January–February		March–May		June–September		October–December	
			Pre-COVID		1st peak		Recovery		2nd peak
		2018/2019	2020	2018/2019	2020	2018/2019	2020	2018/2019	2020
		*N* (%)	*N* (%)	*N* (%)	*N* (%)	*N* (%)	*N* (%)	*N* (%)	*N* (%)
Total		1213	551	1797	640	2229	999	1629	799
Age	< 55 years	142 (12)	84 (15) *	220 (12)	96 (15)	284 (13)	169 (17) *	195 (12)	97 (12)
	55–75 years	727 (60)	296 (54)	1046 (58)	344 (54)	1264 (57)	491 (49)	916 (56)	486 (61)
	> 75 years	344 (28)	171 (31)	531 (30)	200 (31)	681 (31)	339 (34)	518 (32)	216 (27)
Gender	Male	764 (63)	358 (65)	1103 (61)	391 (61)	1390 (62)	605 (61)	1043 (64)	480 (60)
	Female	449 (37)	193 (35)	694 (39)	249 (39)	839 (38)	394 (39)	586 (36)	319 (40)
PS	0	460 (62)	255 (66)	665 (59)	272 (61)	923 (61)	401 (57) *	641 (60)	386 (69) *
	1	209 (28)	95 (24)	354 (31)	115 (26)	424 (28)	245 (35)	314 (30)	130 (23)
	2–4	69 (9)	29 (10)	115 (10)	57 (13)	158 (11)	60 (9)	109 (10)	45 (8)
	Unknown	475	162	663	196	724	293	565	238
Clinical	I	273 (24)	156 (29)	443 (27)	152 (25) *	513 (25)	221 (23)	414 (27)	217 (29)
Stage	II	223 (20)	93 (18)	315 (19)	95 (16)	416 (20)	175 (18)	319 (21)	151 (20)
	III	435 (38)	188 (36)	618 (37)	224 (37)	775 (37)	352 (37)	541 (35)	248 (33)
	IV	202 (17)	92 (17)	289 (17)	142 (23)	389 (19)	206 (22)	257 (17)	145 (19)
	Unknown	80	22	132	27	136	45	98	38

*P*-values were calculated using Chi-squared tests, asterisks indicate statistically significant differences (*p* < 0.05)

*PS* performance status

The proportion of patients aged 55–75 years was significantly lower during the 1st peak and recovery period of 2020 compared to 2018/2019 for colon cancer patients, and significantly lower during pre-COVID and recovery period of 2020 compared to 2018/2019 in rectal cancer patients. The PS for colon and rectal cancer patients differed: worse PS in colon cancer patients during the 1st peak and recovery period of 2020 and better PS in rectal cancer patients during the recovery and 2nd peak of 2020 compared to 2018/2019. Furthermore, the stage distribution differed significantly in some periods compared to 2018/2019. For colon cancer, the proportion of early stages (I and II) was lower in the 1st peak and recovery period; for rectal cancer, the proportion of early stages was lower in the 1st peak.

### Time interval between diagnosis and initial treatment

**Fig. 1:**
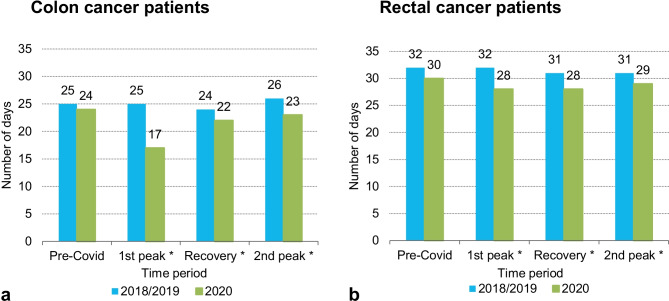
Median time intervals between diagnosis and initial treatment for colon (**a**) and rectal (**b**) cancer patients by period of 2018/2019 and 2020. Pre-Covid, January-February; 1^st^ peak, March-May; Recovery, June-September; 2^nd^ peak, October-December. *P*-values were calculated using Mann-Whitney U tests, asterisks indicate statistically significant differences (*p *<0.05)

Median time intervals between diagnosis and initial treatment are presented in Fig. 1A (colon cancer), B (rectal cancer). Both in colon and rectal cancer patients, the median time intervals between diagnosis and initial treatment was on average 4 days shorter in COVID periods of 2020 compared to those periods of 2018/2019. In colon cancer patients diagnosed in 2020, median time interval increased from 17 days in the 1st peak to 23 days in the 2nd peak, which is comparable to the pre-COVID period. In rectal cancer patients diagnosed in 2020, median time intervals did not increase over time.

### Time interval between (neo) adjuvant therapy and surgery

**Fig. 2:**
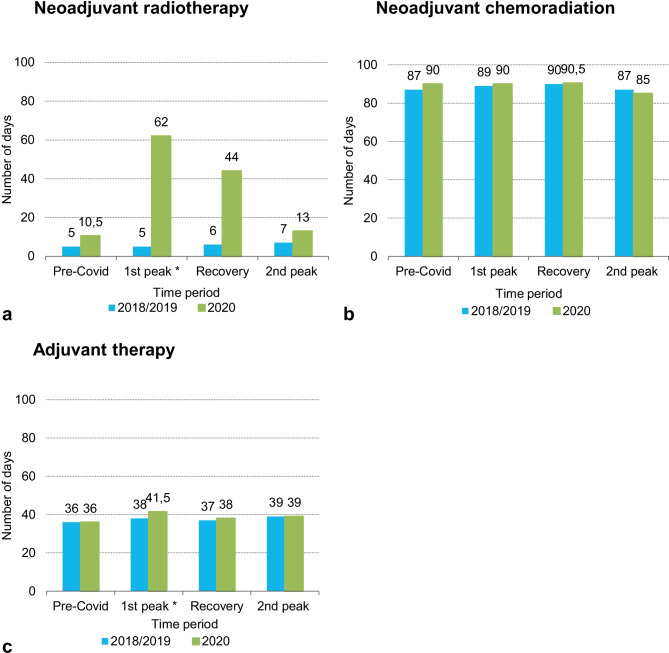
Median time interval between neoadjuvant radiotherapy and surgery (**a**), between neoadjuvant chemoradiation and surgery for clinical stage II and III rectal cancer patients (**b**) and between surgery and adjuvant therapy for stage III colon cancer patients (**c**). Pre-Covid, January-February; 1^st^ peak, March-May; Recovery, June-September; 2^nd^ peak, October-December. *P*-values were calculated using Mann-Whitney U tests, asterisks indicate statistically significant differences (*p *<0.05)

In clinical stage II and III rectal cancer patients, the median time interval between neoadjuvant radiotherapy and surgery was longer in the 1st peak and recovery period of 2020 compared to corresponding months of the years 2018/2019: 62 days and 44 days in 2020 versus 5 days and 6 days in 2018/2019 (Fig. 2A). However, the difference in median time between recovery period of 2020 and the same period of 2018/2019 was not statistically significant. Moreover, no significant differences were found among the different periods regarding the median time interval between neoadjuvant chemoradiation and surgery (Fig. 2B). In stage III colon cancer patients, median time interval between surgery and adjuvant therapy was longer in the 1st peak of 2020 compared to 2018/2019: 41.5 days in 2020 versus 38 days in 2018/2019 (Fig. 2C).

### Treatment

The proportions of patients with (clinical) stage I–III colorectal cancer who underwent (acute) resection or who received a stoma by period are shown in Table 3 (colon cancer) and Table 4 (rectal cancer).

**Table 3:** Number of rectal cancer patients with clinical stage I-III who underwent (acute) resection or received a stoma in pre-COVID, 1st peak, recovery period, and 2nd peak of 2020 versus 2018/2019

		January–February		March–May		June–September		October–December	
			Pre-COVID		1st peak		Recovery		2nd peak
		2018/2019	2020	2018/2019	2020	2018/2019	2020	2018/2019	2020
		*N* (%)	*N* (%)	*N* (%)	*N* (%)	*N* (%)	*N* (%)	*N* (%)	*N* (%)
Total		2771 (100)	1136 (100)	4060 (100)	1249 (100)	5186 (100)	2135 (100)	3794 (100)	1725 (100)
Resection	No surgery	203 (7)	108 (10)	293 (7)	120 (10)*	388 (7)	217 (10)	275 (7)	150 (9)
	Local surgery only	318 (11)	99 (9)	408 (10)	91 (7)	454 (9)	156 (7)	355 (9)	136 (8)
	Surgery	2250 (81)	929 (82)	3359 (83)	1038 (83)	4344 (84)	1762 (83)	3164 (84)	1439 (83)
	Elective	2068 (92)	856 (92)	3054 (91)	908 (88)*	3959 (92)	1569 (89)	2886 (91)	1312 (91)
	Acute < 12 h	109 (5)	46 (5)	183 (5)	74 (7)	235 (5)	111 (6)	168 (5)	69 (5)
	Acute > 12 h	66 (3)	26 (3)	108 (3)	54 (5)	133 (3)	79 (4)	101 (3)	54 (4)
Stoma		391 (14)	131 (12)	540 (13)	178 (14)	742 (14)	301 (14)	516 (14)	195 (11)

Pre-COVID, January–February; 1st peak, March–May; Recovery, June–September; 2nd peak, October-December

*P*-values were calculated using Chi-squared tests, and asterisks indicate statistically significant differences (*p* < 0.05)

**Table 4:** Number of colon cancer patients with stage I-III who underwent a (acute) resection or received a stoma in pre-COVID, 1st peak, recovery period, and 2nd peak of 2020 versus 2018/2019

		January–February		March–May		June–September		October–December	
			Pre-COVID		1st peak		Recovery		2nd peak
		2018/2019	2020	2018/2019	2020	2018/2019	2020	2018/2019	2020
		*N* (%)	*N* (%)	*N* (%)	*N* (%)	*N* (%)	*N* (%)	*N* (%)	*N* (%)
Total		1011 (100)	458 (100)	1508 (100)	498 (100)	1839 (100)	791 (100)	1371 (100)	654 (100)
Resection	No surgery	174 (17)	81 (18)	289 (19)	96 (19)	383 (21)	154 (19)	268 (20)	134 (20)
	Local surgery only	100 (10)	48 (10)	146 (10)	46 (9)	186 (10)	74 (9)	165 (12)	85 (13)
	Surgery	737 (73)	329 (72)	1073 (71)	356 (71)	1270 (69)	563 (71)	938 (68)	435 (67)
	Elective	728 (100)	328 (100)	1056 (99)	355 (100)	1265 (100)	557 (99)	929 (99)	428 (99)
	Acute < 12 h	1 (0)	1 (0)	4 (0)	0 (0)	3 (0)	2 (0)	2 (0)	4 (1)
	Acute > 12 h	1 (0)	0 (0)	3 (0)	0 (0)	0 (0)	1 (0)	3 (0)	2 (0)
Stoma		503 (50)	206 (45)	713 (47)	240 (48)	859 (47)	382 (48)	630 (46)	277 (42)

*P*-values were calculated using Chi-squared tests

First of all, the proportion of colon cancer patients that did not undergo surgery was higher during the pre-COVID period and 1st peak of 2020 compared to the same periods of 2018/2019. Moreover, a lower proportion of colon cancer patients underwent elective surgery during the 1st peak of 2020 compared to the same period of 2018/2019, 88% in 2020 versus 91% in 2018/2019, respectively. Furthermore, the proportion of colon cancer patients receiving a stoma was lower during the pre-COVID period and 2nd peak of 2020 compared to the same periods of 2018/2019, 12% and 11% in 2020 versus 14% for both periods in 2018/2019, respectively. No significant differences were found in rectal cancer patients undergoing (acute) resection or receiving a stoma.

The proportion of stage III colon cancer patients receiving adjuvant therapy varied between 57 and 64% in the defined periods of 2020, and the proportion of clinical stage II-III rectal cancer patients receiving neoadjuvant therapy varied between 67 and 75% in the periods of 2020. Comparing those proportions per period between 2018/2019 and 2020 showed no significant differences.

No significant differences were found regarding proportion of CRC patients with stage IV who received systemic therapy or who did not receive anti-cancer therapy in all time periods (data not shown).

## Discussion

This nationwide study examined the effects of the COVID-19 pandemic on CRC care in the Netherlands in 2020. We observed only minor changes, mostly during the 1^st^ peak period of 2020. Our findings suggest that the COVID-19 pandemic had limited impact on CRC care in the Netherlands.

From mid-March 2020 to mid-May 2020, during the 1st peak of COVID-19 infections, the screening program for CRC in the Netherlands was suspended. Since the age group eligible for CRC screening is 55–75 years in the Netherlands and almost half of the screen-detected tumors are stage I tumors [5, 6, 13], we found a lower proportion of patients aged 55–75 years and a higher proportion of stage IV CRC patients during the 1st peak period of 2020. Due to this suspension, mainly fewer stage I tumors were diagnosed and this shifted the proportions of the stage distribution. As the restart of the CRC screening program has gradually built up to pre-pandemic levels from July 2020 to September 2020 [8], we also found a lower proportion of CRC patients aged 55–75 and a higher proportion of stage IV colon cancer patients during the recovery period of 2020. Due to the temporary halt of the screening program, less asymptomatic tumors were found, which is in line with results of previous studies [5, 6, 8].

The large number of COVID-19 infections in 2020 in the Netherlands led to a situation where demand on hospitals and intensive care units (ICU) increased enormously. Hence, hospitals had a limited overall surgical capacity and elective surgeries were postponed during the pandemic [6]. Results of various studies show that CRC treatment was significantly delayed during the COVID-19 pandemic. In the UK, for example, treatment was delayed or canceled in 23.4% of CRC cases [14]. In addition, an international review in 84 countries showed treatment delay in 70.9% of CRC cases [15]. Contrary to expectations, our study observed a significantly shorter interval between diagnosis and initial treatment in CRC patients during the COVID-19 periods. This might partially be explained by the decrease in (colorectal) cancer diagnoses [6]. In addition, postoperative ICU admission was a limiting factor for surgical procedures in many hospitals during the pandemic, but is normally not required for CRC surgery. The median time between diagnosis and initial treatment was lower during 1^st^ peak period in 2020 compared to previous years. Moreover, the median time during the other periods of 2020 remained lower compared to the same periods of 2018/2019. However, the median times by period in 2020 increased over time. A possible explanation for this finding might be the growing number of CRC diagnoses because of the restart of the CRC screening program. Moreover, surgical procedures have been re-started in a stepwise way, partly due to the uncertain situation where it was not known whether capacity would still be available a week later. Therefore, less operation room capacity was available for elective CRC procedures because of the re-start of for example cardiac and neurosurgery procedures.

Results of our study showed a significantly longer interval between neoadjuvant radiotherapy and surgery and between surgery and adjuvant therapy, only during the 1st peak period of 2020. It is difficult to distinguish whether differences in median time between (neo)adjuvant therapy and surgery is COVID-19 related or a result of the change in clinical practice. Differences might be partly explained by the recommendations to postpone surgery after neoadjuvant radiotherapy and to postpone adjuvant therapy after surgery as published by the Dutch Multidisciplinary Oncology Foundation (SONCOS) and the Dutch Association for Medical Oncology (NVMO) in the beginning of March 2020. Since the median time between (neo)adjuvant therapy and surgery showed no significant differences for the periods after the 1st peak period, the difference in median time found during 1st peak period in this study is probably COVID-19 related. The large difference in median time during recovery period of 2020 is probably not significant due to a low number of patients included in this group. Therefore, the difference in median time might not be COVID-19 related, and could be a result of a change in clinical practice, namely to wait longer after neoadjuvant therapy and to further delay surgery to increase the clinical response rate [16].

Studies in various countries have shown a significant decrease in CRC surgery during the COVID-19 pandemic. The rate of this decrease was 51% in Poland [17], 40% in India [18], and 34% in Germany [19]. Also for breast cancer treatment in the Netherlands significant differences in chemotherapy, mastectomy with immediate reconstruction and primary hormonal treatment are reported [20]. Previous research based on data from 25 hospitals in the Netherlands found minimal effects during the 1^st^ peak of 2020 [10]. The current nationwide data confirm these findings and therefore the setbacks in treatment of CRC during the pandemic was limited in the Netherlands. This was probably also related to the fact that the screening capacity was 20% increased, which increased the early detection of CRC having the less invasive treatment options.

## Conclusion

This was the first study using nationwide data from the NCR for all patients diagnosed with CRC in the Netherlands to investigate the impact of the COVID-19 pandemic on CRC care. Only minor changes in the care for CRC patients were revealed during the COVID-19 pandemic, mostly during the 1st peak period. These changes were partly due to a higher proportion of patients presenting with symptoms during the pandemic, as a result of the suspension of the national screening program. However, some changes could be caused by adjustments in CRC care, irrespective of the pandemic. In conclusion, the impact of the COVID-19 pandemic on CRC care in the Netherlands was found to be limited and CRC care for patients in the Netherlands has continued, despite the unprecedented impact of the COVID-19 pandemic on global healthcare. However, long-term effects cannot be precluded. Therefore, future studies will investigate the long-term effects of the COVID-pandemic on the colorectal cancer care.
